# Supplementary material for: Association of weaning preparedness with extubation outcome of mechanically ventilated patients in medical intensive care units: a retrospective analysis
Source: PeerJ. 2020 Apr 13;8:e8973. doi: 10.7717/peerj.8973 (PMC7161570; doi:10.7717/peerj.8973)
Supplement: Supplemental Information 2 — Description and Table summarizing sensitivity analysis separating ’wakefulness’ from the original WEANSNOW scoring. [file peerj-08-8973-s002.docx]

**Association of weaning preparedness with extubation outcome of mechanically ventilated patients in medical intensive care units: a retrospective analysis**

**RESULTS**

For sensitivity analysis, we separated the item ‘wakefulness’ from the WS score in the multivariate analysis. Logistic regression showed that the ‘total WS minus the wakefulness score 1’ (designated as ‘WEANSNO score’ in Table 1S) remained a significant associating factor for extubation failure (OR=3.552, 95% CI=1.548-8.150, p=0.003), along with intubation duration > 21 days (OR=7.862, 95% CI=3.507 -17.623, p<0.001). In contrast, wakefulness was not independently associated with extubation failure in this study group (OR=1.372, 95% CI=0.630-2.991, p=0.426) (Table 1S).

**TABLE**

**Table 1S**. **Multivariate logistic regression analysis of the factors and variables associated with extubation failure**

| Variable | N (%) | OR | 95% CI | p-value |
| --- | --- | --- | --- | --- |
| Heart failure | 75 (36.6%) | 0.589 | 0.265-1.308 | 0.193 |
| Type 1 or Type 2 respiratory failure | 143 (69.8%) | 0.533 | 0.257-1.191 | 0.130 |
| Intubation days > 21 | 53 (25.9%) | 7.862 | 3.507-17.623 | <0.001 |
| MIP ≤ -20 cmH_2_O | 189 (92.2%) | 0.655 | 0.158-2.710 | 0.559 |
| MEP ≥ 30 cmH_2_ O | 157 (76.6%) | 1.198 | 0.460-3.122 | 0.711 |
| RSBI<105 | 26 (13.3%) | 0.487 | 0.161-1.473 | 0.203 |
| WEANS NO score ≥ 1 | 74 (36.1%) | 3.552 | 1.548-8.150 | 0.003 |
| ‘Wakefulness’ score=1 | 68 (33.2%) | 1.372 | 0.630-2.991 | 0.426 |

MIP: maximum inspiratory pressure; MEP: maximum expiratory pressure; RSBI: rapid shallow breathing index; OR: odds ratio; CI: confidence interval
